# Ultra-processed foods consumption associated with food addiction in Chilean young adults

**DOI:** 10.3389/fnut.2025.1722589

**Published:** 2026-01-08

**Authors:** Ximena Díaz-Torrente, Carina Valenzuela, Michelle Valenzuela, Ashley N. Gearhardt

**Affiliations:** 1 Carrera de Nutrición y Dietética, Facultad de Medicina, Universidad del Desarrollo, Santiago, Chile; 2 Escuela de Nutrición y Dietética, Facultad de Farmacia, Universidad de Valparaíso, Valparaíso, Chile; 3 Centro de Micro-Bioinnovación (CMBi), Centro de Investigación del Comportamiento Alimentario (CEIC), Escuela de Nutrición y Dietética, Facultad de Farmacia, Universidad de Valparaíso, Valparaíso, Chile; 4 Department of Psychology, University of Michigan, Ann Arbor, MI, United States

**Keywords:** food addiction, NOVA score, ultra-processed foods, YFAS 2.0-Chile, young adults

## Abstract

**Background:**

Ultra-processed foods (UPF), most of which have hyper-palatable properties, have been implicated in overconsumption and adverse health outcomes, yet their association with food addiction (FA) remains underexplored in young adults.

**Aim:**

To assess the association between marker foods for UPF consumption and FA in Chilean university students, comparing UPF consumption among individuals with and without FA.

**Methods:**

In a cross-sectional study, 287 young adults (median age: 21 years) completed the NOVA Screener -which captures the presence and number of UPF items consumed during the previous day- and the Chilean version of the Yale Food Addiction Scale 2.0 (YFAS 2.0-Chile). Anthropometric measurements and body composition were obtained for 271 participants. Logistic regression models were adjusted for gender, physical activity, and Body Mass Index (BMI).

**Results:**

The prevalence of FA was 17.4%, predominantly severe cases. Participants with FA had significantly higher median NOVA scores for beverages, snacks, and total UPF consumption. FA was positively associated with greater consumption of UPF beverages (OR 1.48, *p* = 0.014), snacks (OR 1.26, *p* = 0.044), and NOVA score (OR 1.12, *p* = 0.042). Individuals with FA also exhibited higher BMI and body fat percentage.

**Conclusion:**

These findings highlight a significant association between UPF consumption and FA in young adults, suggesting that UPF consumption may potentially contribute to addictive eating behaviors and associated metabolic risks. Public health strategies targeting UPF consumption may be essential in preventing FA and related health conditions.

## 1 Introduction

Ultra-processed foods (UPF), defined under the NOVA classification system are extensively modified through physical, biological, and chemical processes, resulting in products with high energy density, added sugars, saturated and trans fats, and sodium (1). The NOVA classification, briefly, categorizes foods according to the extent and purpose of industrial processing, ranging from unprocessed or minimally processed foods to UPF, which are formulations primarily composed of substances derived from foods and additives designed to enhance sensory properties (2). These compositional and processing characteristics enhance the palatability of UPFs, making them highly attractive and promoting overconsumption (2–4). Evidence from longitudinal studies has established associations between UPF consumption and obesity, as well as related health issues, such as cardiovascular risk factors and metabolic syndrome (5–8). Furthermore, experimental research has demonstrated that diets based predominantly on UPFs lead to excessive caloric intake and weight gain, largely due to their widespread availability and addictive properties (9). This overconsumption surpasses energy requirements and contributes to the global obesity epidemic (10). Most studies assessing UPF consumption use tools such as the 24-h dietary (11–14) reminder that require experienced interviewers, time and willingness on the part of the participants. On the other hand, the recorded foods then need to be classified according to NOVA, which also requires trained people for classification. A novel instrument, the NOVA Screener, is a brief questionnaire designed to identify markers of UPF consumption based on food categories, offering a practical alternative to time-intensive dietary assessment methods. The NOVA Screener has been developed to simplify this process, even by applying it through mobile phones, tablets or computers (15).

Despite the recognized health risks of UPFs, few studies have examined their association with disordered eating or appetite dysregulation, particularly in young adult populations (16). The concept of food addiction (FA), particularly to hyper-palatable UPFs, has been proposed as a potential mechanism underlying overeating and subsequent obesity (17). Neuroimaging evidence shows that individuals with FA exhibit heightened activation in reward-related regions such as the striatum and medial orbitofrontal cortex when exposed to cues of hyper-palatable or UPF. These activation patterns resemble those observed in substance use disorders, suggesting that FA—rather than obesity per se—engages reward mechanisms that are particularly sensitive to the reinforcing properties of UPFs (18–20). This shared neurobiological framework suggests that FA—rather than obesity per se—may involve reward-related mechanisms similar to those implicated in substance use disorders (21, 22) highlights the need for further investigation into FA and its implications for public health, particularly in vulnerable populations such as young adults. Young adulthood, particularly within the university environment, represents a critical period for the establishment of eating behaviors, as stress, irregular schedules, and campus food environments can strongly influence diet quality and eating patterns (23, 24). In addition, young adulthood is a critical stage for the development of eating behaviors that will persist in adulthood (25). Detecting altered eating behaviors in this period could have positive consequences for health outcomes.

The aim of the present study was to compare UPF consumption in young Chilean adults with and without FA, as well as to evaluate the association between UPF consumption and FA, contributing to the understanding of the associations between UPF consumption, eating behaviors, and health outcomes.

## 2 Methods

### 2.1 Design, participants and procedure

A cross-sectional study was conducted in a non-probabilistic convenience sample during the months of August to September 2023. The sample was composed of young adults (university students) who were recruited from two Universities from two different regions of Chile (Metropolitan Region and Valparaíso Region). The inclusion criterion was subjects over 18 years, who were not on medication for weight loss because of the effects on appetite inhibition. In addition, pregnant, or breastfeeding women were excluded.

The participants were invited to participate in this study when they were walking near the private area of measurements installed on both university campuses. Those who were interested in participating came to the measurement area voluntarily and were given informed consent. Participants who decided to participate signed an informed consent. Once in the measurement area, they were asked to complete a questionnaire about demographic data; whether they engage in physical activity; whether they live alone; whether they were under psychological treatment; after this questionnaire, they were given the NOVA screener and then YFAS 2.0-Chile. Once these scales were completed, anthropometric and body composition measurements were carried out by trained research assistants. The participants were told that participation in the study was voluntary, without financial compensation, but that they would obtain their anthropometric measurements and nutritional diagnosis.

### 2.2 Measures

#### 2.2.1 Demographic data

Participants were asked to provide information about gender, age, physical activity (yes/no), living alone (yes/no) – given its potential influence on eating behaviors among young adults-, and psychological treatment (yes/no).

#### 2.2.2 Anthropometric measurements and body composition

Weight and height were measured using a SECA^®^ brand scale (0.1 kg precision) and a height rod (0.1 cm precision). The nutritional status was determined by calculating the Body Mass Index (BMI). It was classified according to the criteria of the World Health Organization as low weight (BMI ≤ 18.5), normal nutritional status (BMI 18.5–24.9 kg/m^2^), overweight (BMI 25.0–29.9 kg/m^2^), or obesity (BMI ≥ 30 kg/m^2^) (26). Waist circumference (WC) was measured using a flexible tape measured at the midpoint between the iliac crest and the last rib. The participant remained standing with the arms next to the body and the trunk free of clothing. The measurement was made with the abdomen relaxed at the end of expiration (27). The InBody^®^ technology BIA was used to measure body composition, determining the percentage of body fat mass (%BF) and lean mass (28). Trained and standardized evaluators performed the measurements. The privacy of the participants was protected during the anthropometric measurements.

### 2.3 NOVA screener for the consumption of ultra-processed foods

The NOVA Screener is an abbreviated questionnaire recently developed to estimate UPF consumption; it asks about the consumption of items from specific groups of UPF and beverages during the day prior to the interview without inquiring about the quantity consumed. The presence of any of these foods and beverages yields a score of one and the absence scores zero. The NOVA Score then represents the sum of these scores. The NOVA Screener was developed to include the UPF subgroups with the highest participation in the daily energy intake of the Brazilian population. Furthermore, the NOVA Score showed substantial agreement with the UPF dietary share obtained 24-hR in the Brazilian adults (29). The instrument applied in the Brazilian population presents a list of 23 UPF subgroups representing three categories: beverages (six groups), products that replace or accompany meals (10 groups), and products often consumed as snacks (seven groups). As reported by the group of researchers who developed it, NOVA Screener is being adapted for use in India, Senegal, and Ecuador, allowing other countries to study the performance of the NOVA score (15). The instrument applied in the Chilean young adults included a list of 20 UPF subgroups representing three categories: beverages (five groups), products that replace or accompany meals (eight groups), and products often consumed as snacks (seven groups) (see Supplementary material). The questionnaire asks if the person consumed each of the 20 UPFs listed in the past 24 h and assigns one point if a product of the subgroup was consumed and zero otherwise. The result is expressed as a Nova score of UPF consumption going from 0 if the young adults did not consume any of the products to 20 if the young adults consumed one of each type of 20 UPF subgroups from the list. NOVA screener has already been used previously in the Chilean population (30), and has proven to be a good predictor of UPF consumption compared to UPF data obtained through a 24-h dietary recall in a sample of Chilean preschool children (31).

### 2.4 Chilean version of YFAS 2.0

The YFAS 2.0 is a 35-item self-report scale scored on an eight-level Likert scale (from 0 = never to 7 = every day), designed by Gearhardt et al. (32) to assess FA symptoms over the previous 12 months, based on 11 diagnostic criteria for substance-related and addictive disorders proposed in the DSM-5 (33). These scorings produce two measurements: (a) a continuous symptom count score that reflects the number of fulfilled diagnostic criteria (ranging from 0 to 11); and (b) a FA threshold based on the number of symptoms (at least 2) and self-reported clinically significant impairment or distress. This final measurement allows the dichotomous classification of FA (FA vs. No FA). Based on the revised DSM-5 taxonomy, the YFAS 2.0 also provides severity cutoffs for patients surpassing the threshold for FA: mild (2–3 symptoms), moderate (4–5 symptoms), and severe (6–11 symptoms). In the current study YFAS 2.0-Chile was applied. This instrument showed good internal consistency (Kuder–Richardson’s *α* of 0.85 and McDonald’s *ω* of 0.88) and good fit indices in the factor analysis (CFI = 0.988, TLI = 0.985, SRMR = 0.063, RMSEA = 0.040, with all factor loadings greater than 0.69) in the validation study (34).

### 2.5 Statistical analysis

Stata statistical software version 17.0 was used for all statistical analyses (StataCorp, LLC. Texas, United States). A total of 287 participants completed both questionnaires (NOVA Screener and YFAS 2.0-Chile); however, 16 participants lacked anthropometric measurements due to scheduling conflicts or personal refusal to undergo body composition assessment. Descriptive statistics are presented as median and interquartile range – because of the distribution- or number and percentage dependent on variable type. The *U* Mann Whitney test was used to explore differences in the median of NOVA Score category (beverages, products that replace or accompany meals, snacks) between participants classified with FA (FA vs. No FA).

Logistic regression models were used to explore the association between NOVA Score category with classification of FA (FA vs. No FA). All models were controlled for age, gender, living alone, be undergoing psychological treatment, and BMI. Statistical significance was set at *p* < 0.05.

## 3 Results

The sample studied included 287 young adults with a median age of 21 years. FA prevalence was 17.4%, with severe FA representing 82.0% of cases. The most frequently reported symptom was consuming food in larger quantities or over longer periods than intended (33.1%), followed by a persistent desire or unsuccessful efforts to reduce consumption (28.2%) (Supplementary Table S1).

Demographic and anthropometric characteristics of the sample revealed significant differences between young adults with FA and No FA. Among participants meeting the criteria for FA, women predominated (78.0%) compared to men (22.0%; *p* < 0.001) and were less likely to be physically active (44.0%) than those without FA (70.9%; *p* < 0.001). Additionally, most participants with FA were classified as overweight or obese (37.5 and 18.8%, respectively), compared to participants without FA, which mostly presented a healthy BMI category (58.3%) (Table 1).

**Table 1:** Differences in demographic and anthropometric characteristics of young adults with food addiction or no food addiction.

Measure or category		Total sample (*n* = 287)	FA (*n* = 50)	No FA (*n* = 237)	*p*-value
Age, median (IQR)	Years	21 (20–22)	20.5 (20–22)	21 (20–23)	0.421*
Gender, % (*n*)	Female	55.8 (160)	78.0 (39)	51.1 (121)	**<0.001** ^**¥**^
	Male	44.2 (127)	22.0 (11)	48.9 (116)	
Physical activity, % (*n*)	Yes	66.2 (190)	44.0 (22)	70.9 (168)	**<0.001** ^**¥**^
	No	33.8 (97)	56.0 (28)	29.1 (71.1)	
Psychological treatment, % (*n*)	Yes	18.5 (53)	24.0 (12)	17.3 (41)	0.267^¥^
	No	81.5 (234)	76.0 (38)	82.7 (196)	
Living alone, % (*n*)	Yes	16.7 (48)	20.0 (10)	16.0 (38)	0.495^¥^
	No	83.3 (239)	80.0 (40)	84.0 (199)	
Weight, median (IQR)	Kg	65.3 (56.6–76.7)	66 (57.1–80.5)	65.2 (56.6–76.4)	0.500*
		(*n* = 271)	(*n* = 48)	(*n* = 223)	
Body Mass Index, median (IQR)		24.1 (21.4–26.7)	25.4 (22.9–27.8)	23.6 (21.3–26.6)	0.028*
BMI category, % (*n*)	Underweight	3.3 (9)	4.2 (2)	3.1 (7)	**0.024** ^**¥**^
	Healthy weight	55.0 (149)	39.6 (19)	58.3 (130)	
	Overweight	32.8 (89)	37.5 (18)	31.8 (71)	
	Obesity	8.9 (24)	18.8 (9)	6.7 (15)	
Waist circumference, median (IQR)	Cm	77.5 (70.6–84.0)	77.1 (71.5–86.7)	77.5 (70.5–84.0)	0.441*
Body fat, median (IQR)	%	31.3 (23.0–37.4)	37.1 (30.5–41.2)	28.9 (21.1–35.9)	<0.001*
Lean Mass, median (IQR)	Kg	24.6 (20.2–31.6)	22.5 (19.0–28.1)	25.4 (20.3–31.9)	0.040*

FA, food addiction; No FA, no food addiction. ^¥^Chi2 or *U Mann–Whitney test. In bold type *p* < 0.05.

Given the well-established sex-related differences in body composition, an exploratory analysis was conducted separating these measures by gender. As expected, women exhibited a smaller waist circumference, a higher percentage of body fat, and a lower lean mass compared to men. To account for these differences, a gender-stratified exploratory analysis was performed, and the detailed results of this analysis are provided in the Supplementary material. In men, individuals with FA showed significantly higher WC and BF% compared to No FA, with no differences observed in lean mass. In women, no differences were found in WC and lean mass, but BF% was significantly higher in those with FA (Supplementary Table S2).

The frequency of consumption of UPF subgroups showed that beverages like instant coffee or tea (67.6%) and flavored yogurt (42.9%) were the most consumed items. Among snacks, sweet biscuits (50.2%) and chocolate bars (26.8%) were highly prevalent (Table 2).

**Table 2:** Frequency of consumption of every ultra-processed food subgroup of NOVA Screener.

Category NOVA Screener	Ultra-processed food subgroups	Frequency of consumption (%)
Beverages	Regular or non-caloric sodas (sugared and sugar-free)	46.0
	Canned or bottled fruit juice or powdered drink mix	41.8
	Flavored milk	29.3
	Tea, instant coffee from a machine, or prepared from powder	67.6
	Any flavored yogurt	42.9
Products that replace some food or meal	Sausage, hamburger, or nuggets	22.3
	Ham, salami, or mortadella	42.5
	Loaf or packaged bread	58.4
	Frozen French fries or fast-food chain fries	20.6
	Margarine, mayonnaise, or ketchup	47.0
	Instant noodles or packaged soup	23.3
	Lasagna or other frozen ready-made meals	5.9
	Salad dressings or vinaigrettes (ready-to-eat)	8.7
Snacks	Packaged salty snacks (chips or crackers)	23.7
	Sweet biscuits with or without filling	50.2
	Packaged cake	29.6
	Cereal bar	19.9
	Ice cream or packaged frozen dessert	19.5
	Chocolate bars and chocolates	26.8
	Breakfast cereal	19.9

Young adults with FA had significantly higher median scores for beverages (3 IQR 2–3 vs. 2 IQR 1–3, *p* = 0.027), snacks (2 IQR 1–3 vs. 2 IQR 1–2, *p* = 0.009), and overall, NOVA Score (7 IQR 5–8 vs. 6 IQR 4–8, *p* = 0.032) compared to non-FA young adults (Table 3).

**Table 3:** Differences in the NOVA Score of the NOVA Screener categories between young adults with food addiction or no food addiction.

NOVA Screener category	Total sample (*n* = 287)	FA (*n* = 50)	No FA (*n* = 237)	*p*-value
Beverages	2 (2–3)	3 (2–3)	2 (1–3)	**0.027**
Products	2 (1–3)	2 (1–3)	2 (1–3)	0.888
Snacks	2 (1–3)	2 (1–3)	2 (1–2)	**0.009**
NOVA Score	6 (5–8)	7 (5–8)	6 (4–8)	**0.032**

FA, food addiction; No FA, no food addiction. Values are presented in the median (IQ range). *U* Mann–Whitney test. In bold type *p* < 0.05.

Logistic regression analyses showed that FA was significantly associated with higher consumption of beverages (OR 1.36, *p* = 0.026) and snacks (OR 1.24, *p* = 0.035) in unadjusted models. These associations remained significant in adjusted models, with FA also linked to higher NOVA Scores (OR 1.12, *p* = 0.037) (Table 4).

**Table 4:** Logistic regression of food addiction and NOVA Score of the NOVA Screener categories in young adults.

Food addiction	Unadjusted model (*n* = 287)			Adjusted model (*n* = 271)		
NOVA Screener category	OR	CI 95%	*p*-value	OR	CI 95%	*p*-value
Beverages	1.36	1.03–1.79	**0.026**	1.50	1.09–2.06	**0.012**
Products	0.97	0.80–1.18	0.811	1.03	0.84–1.28	0.727
Snacks	1.24	1.02–1.53	**0.035**	1.26	1.00–1.58	**0.043**
NOVA Score	1.08	0.98–1.19	0.095	1.12	1.00–1.25	**0.037**

OR, odds ratio. Model adjusted for gender, physical activity, living alone, psychological treatment, and BMI. In bold type *p* < 0.05.

To verify that the use of Nova Screener categories as continuous variables in the logistic regression model was appropriate, these components were classified into tertiles. A significant linear trend was observed for UPF beverages (*χ*^2^ = 7.44, *p* = 0.006) and snacks (*χ*^2^ = 4.39, *p* = 0.036), with no evidence of quadratic effects, supporting a monotonic increase in AF risk at all levels of consumption. No significant trend was observed for UPF foods (*p* = 0.67). The total NOVA score showed a positive linear trend, although not significant (*p* = 0.10). These findings support the modeling of NOVA components—and the total score—as continuous predictors in the main analyses (Supplementary Table S3).

Interaction terms between sex and each NOVA Screener category (foods, beverages, and snacks) were tested to assess whether the association between UPF consumption and FA differed by gender. None of the interaction terms were statistically significant (sex × foods: *p* = 0.96; sex × beverages: *p* = 0.34; sex × snacks: *p* = 0.15), indicating that the relationship between UPF exposure and FA was similar for men and women (data no showed in tables).

Finally, considering that BMI could be a mediating variable rather than a confounding variable, we conducted sensitivity analyses running the regression models both with and without BMI adjustment. The results were highly consistent, with similar effect sizes and significance patterns across models (data no showed in tables).

## 4 Discussion

The current study evaluated FA and the marker foods for UPF consumption from the day before using the NOVA screener, in a sample of young adults. The results identified higher NOVA scores in participants with FA, representing a higher consumption of UPF. The findings of this study reinforce the growing body of evidence indicating that UPF consumption is significantly associated with FA in Chilean young adults.

Our results show a prevalence of 17.4% of FA, a higher value compared to a couple of studies conducted in Chile in non-clinical samples, which showed a prevalence of 10.9 and 10.3%, respectively, (34, 35). Consistent with our results, both studies show a higher FA prevalence in women, suggesting a gender disparity, and support the notion that women might have a greater susceptibility to developing FA (36).

In addition, we found an association between food addiction and nutritional status. Participants diagnosed with FA had a higher BMI than those without FA. Furthermore, most participants with FA were classified as overweight or obese compared to participants without FA, which mostly presented a healthy BMI category, consistent with previous studies showing a higher prevalence of FA in overweight or obese individuals (37–39). This highlights the importance of addressing FA as a risk factor for the development of obesity and other eating-related diseases such as binge-eating disorder, bulimia nervosa, and emotional eating, but also in the treatment of such conditions. Although therapeutic approaches commonly applied in addiction treatment—such as cognitive-behavioral strategies or stimulus control—have been proposed as potentially useful for individuals with FA (40), future longitudinal and clinical research is needed to determine whether such strategies are effective in this population.

The association between FA and higher BMI, as well as an increased % BF, also aligns with previous studies linking FA to obesity and metabolic disorders (38, 41, 42). Given that FA has been associated with impulsive and uncontrolled consumption of energy-dense foods, its presence among young adults suggests that this stage of life may represent a critical period for the establishment of dysfunctional dietary patterns that persist into adulthood, with long-term repercussions on the development of chronic diseases such as type 2 diabetes (43) and cardiovascular conditions (44). In addition, these findings must also be interpreted within the context of university life, where academic stress, irregular schedules, and easy access to UPFs (45) can exacerbate dysregulated eating behaviors and reinforce patterns linked to FA.

Regarding UPF consumption, our findings showed a higher consumption of these foods in individuals with FA, compared to those without FA. Specifically, products with high levels of added sugars, such as instant beverages and flavored yogurts, as well as highly palatable snacks like sweet biscuits and chocolate bars, were the most frequently consumed.

The association between higher consumption of UPF and FA has been reported in other studies. A study conducted in Australia in 2013 and again in 2015 compared UPF intake in 735 young adults between 18 and 35 years of age, with and without FA, using the original version of the YFAS and a food frequency questionnaire coded with the NOVA system for UPF intake. This study reported a 20% prevalence of FA. The daily energy contribution from UPF was significantly higher in participants with FA (39.5 ± 15.5%) than in those without FA (33.0 ± 12.4%) (16).

In another study conducted in Brazil, in adults aged 18–59 years, a prevalence of 18.5% of FA was reported, assessed with the mYFAS 2.0. In addition, individuals with FA had a significantly higher consumption of commonly consumed UPFs, such as hamburgers and/or sausages, instant noodles, packaged snacks and/or crackers, sandwich cookies, sweets and/or candy, compared to individuals without FA (46).

Another study from Brazil showed a 24% prevalence of FA in a sample of 139 children aged 9 to 11 years who were overweight or obese. A frequent consumption of UPFs, specifically cookies/cakes and sausages, was associated with a diagnosis of FA. This study also reported a higher daily energy contribution from UPF among children with FA compared to those without FA, 39% vs. 34%, respectively (47).

These comparisons should be interpreted with caution because Australian and Brazilian studies estimated the energy contribution of UPFs using FFQs or repeated 24-h recalls, whereas our study assessed the presence of UPF subgroups through a brief screener. Although results point in the same direction, the methodological approaches differ, and therefore the comparisons should be viewed as approximate rather than directly equivalent.

UPFs have previously been identified as highly reinforcing in studies on FA due to their impact on brain reward circuits (48–50) and appetite regulation (51). From a clinical perspective, these findings suggest that UPFs may involve neurobiological responses similar to those observed in substance use disorders, promoting compulsive consumption and difficulty in reducing intake (52, 53). This pattern was reflected in the most frequently reported FA symptoms in this study: consuming food in larger quantities or over longer periods than intended, and persistent unsuccessful efforts to reduce intake. This resemblance to substance addiction is consistent with previous research indicating increased activation in brain regions such as the striatum and medial orbitofrontal cortex in individuals with FA (18, 54).

Although NOVA screener does not allow us to evaluate the portion sizes, frequency or dietary share of UPF in people’s diets, it does allow us to evaluate the number of UPF markers present in the diet on the day prior to the survey. The NOVA screener questionnaire has been used previously in Chile to assess UPF consumption in children and its association with the characteristics of the home learning environment during school closures due to the COVID-19 pandemic. In the study, the average NOVA score obtained was 4.3 ± 1.9 SD (30), a lower value than the one reported in this study.

To our knowledge, this is one of the first studies to evaluate UPF consumption using the NOVA screener and its association with FA and nutritional status in Chilean young adults. The strength of this research lies in its contribution to knowledge, providing valuable information that can help to better understand the phenomenon of FA and guide future research and public health policies in Chile and other countries in Latin America, for the development of interventions aimed at promoting healthy eating and preventing obesity and other diet-related diseases.

Certain limitations must be acknowledged, including cross-sectional design, which prevents the establishment of causality. However, the data provides a basis for future longitudinal studies to evaluate how exposure to UPFs during this life stage influences the progression of FA and its metabolic consequences over time. In addition, the NOVA screener was used as a measure of diversity rather than an accurate measure of absolute consumption, which limits our ability to quantify the amount of energy from UPF consumed and estimate nutrient intake.

## 5 Conclusion

Our findings support the hypothesis of an association between FA, nutritional status, and UPF consumption in Chilean university students.

In summary, this study provides evidence that exposure to a greater variety of UPF subgroups is associated with FA in Chilean young adults. These findings underscore the importance of incorporating UPF-related behaviors into public health strategies and highlight the need for longitudinal studies using quantitative dietary assessment methods to clarify causal pathways and inform intervention development.

## Data Availability

The raw data supporting the conclusions of this article will be made available by the authors, without undue reservation.
