# Supplementary material for: Ultra-processed foods consumption associated with food addiction in Chilean young adults
Source: Front Nutr. 2026 Jan 8;12:1722589. doi: 10.3389/fnut.2025.1722589 (PMC12823470; doi:10.3389/fnut.2025.1722589)
Supplement: Supplementary file 1 [file Table_1.DOCX]

**Supplementary material**

Ultra-processed foods consumption associated with food addiction in Chilean young adults.

**Table S1. Food addiction prevalence and symptoms using Chilean YFAS 2.0 in a university student sample.**

| ***Characteristics*** | ***% (n)*** |
| --- | --- |
| Prevalence FA | 17.4 (50) |
| Mild | 4.0 (2) |
| Moderate | 14.0 (7) |
| Severe | 82 (41) |
| Symptom count | 2.5 ± 3.1 |
| ***Criteria*** |  |
| Food consumed in larger quantities or over a longer period than intended | 33.1 |
| Persistent desire or unsuccessful efforts to cut down or control consumption of certain foods | 28.2 |
| Considerable time spent to obtain, consume, or recover from the effects of food | 23.0 |
| Giving up important social, occupational, or recreational activities because of food consumption | 22.3 |
| Continuing to eat certain foods despite physical or psychological problems | 25.4 |
| Tolerance | 12.5 |
| Withdrawal | 25.1 |
| Continued consumption despite social or interpersonal problems | 23.0 |
| Failure to fulfill major role obligation | 13.2 |
| Use in physically hazardous situations | 22.0 |
| Craving | 20.2 |
| Significant distress in relation to food | 22.0 |

**Table S2. Exploratory analysis of body composition measures by food addiction status in men and women.**

| **Gender** | **Body composition measures** | **Food Addiction**  **(n=11)** | **No Food Addiction**  **(n=111)** | **p-value*** |
| --- | --- | --- | --- | --- |
| **Men** | Waist circumference (cm), median (IQR) | 92.5 (86.0 – 101.8) | 81.7 (77.5 – 86.7) | **0.0028** |
|  | Body fat (%), median (IQR) | 30.3 (24.9 – 36.2) | 22.1 (17.5 – 27.7) | **0.0090** |
|  | Lean Mass (kg), median (IQR) | 35.7 (29.1 – 38.0) | 31.9 (28.9 – 34.6) | 0.1343 |
|  |  | **(n=37)** | **(n=112)** |  |
| **Women** | Waist circumference (cm), median (IQR) | 74.2 (71.0 – 80.6) | 71.9 (67.5 – 77.8) | 0.0576 |
|  | Body fat (%), median (IQR) | 38.5 (34.4 – 42.5) | 34.6 (30.6 – 40.4) | **0.0216** |
|  | Lean Mass (kg), median (IQR) | 21.1 (17.7 – 23.1) | 20.3 (18.9 – 22.7) | 0.6477 |

Note: FA: Food Addiction; No FA: No Food Addiction. * U Mann Whitney test. In bold type p<0.05.

**Table S3. Logistic regression models using tertiles of NOVA Screener category and tests for linear trend.**

| **NOVA Screener category** | Tertiles (ref = T1) | OR (95% CI) | p-value | Linear trend (χ², p) | Quadratic (χ², p) |
| --- | --- | --- | --- | --- | --- |
| Beverages | T2 | 1.76 (0.80–3.86) | 0.159 | 7.44, **0.006** | 0.03, 0.865 |
|  | T3 | 3.53 (1.43–8.75) | **0.006** |  |  |
| Products | T2 | 1.30 (0.60–2.78) | 0.505 | 0.18, 0.669 | 0.21, 0.646 |
|  | T3 | 1.22 (0.50–2.98) | 0.668 |  |  |
| Snacks | T2 | 1.83 (0.80–4.18) | 0.152 | 4.39, **0.036** | 0.19, 0.659 |
|  | T3 | 2.44 (1.06–5.61) | **0.036** |  |  |
| NOVA Score | T2 | 1.93 (0.84–4.40) | 0.119 | 2.70, 0.101 | 0.73, 0.394 |
|  | T3 | 2.01 (0.87–4.64) | 0.100 |  |  |

Note: Logistic regression models adjusted for sex, physical activity, and BMI. Linear and quadratic trends tested using polynomial contrast in Stata. Evidence of significant linear trend supports modeling the variable as continuous in main analyses. In bold type p<0.05.

**NOVA Screener for Chile. Abbreviated UPF consumption questionnaire.**

| **Item** | **Question** | **Alternatives** |
| --- | --- | --- |
| Beverages | Which of the following beverages did you drink yesterday? Select all the alternatives that apply. | 1. Regular or noncaloric sodas (sugared and sugar-free)  2. Canned or bottled fruit juice or powdered drink mix  3. Flavored milk  4. Tea, instant coffee from a machine or prepared from powder  5. Any flavored yogurt  0. None of the above options |
| Products | Which of the following foods did you eat yesterday? Select all the alternatives that apply. | 1. Sausage, hamburger, or nuggets  2. Ham, salami, or mortadella  3. Loaf or packaged bread  4. Frozen French fries or fast-food chain fries  5. Margarine, mayonnaise, or ketchup  6. Instant noodles or packaged soup  7. Lasagna or other frozen ready-made meals  8. Ready-made salad sauce (dressing)  0. None of the above options |
| Snacks | Which of the following snacks or desserts did you eat yesterday? Select all the alternatives that apply. | 1. Packaged salty snacks (chips or crackers)  2. Sweet biscuits with or without filling  3. Packaged cake  4. Cereal bar  5. Ice cream or packaged frozen dessert  6. Chocolate bars and chocolates  7. Breakfast cereal  0. None of the above options |
